# Urinary Deposits, Their Diagnosis, Pathology and Therapeutical Indications

**Published:** 1846-02

**Authors:** 


### REVIEWS.

---

**Art. III.**—*Urinary Deposits, their Diagnosis, Pathology, and Therapeutical Indications.* By *Golding Bird, A.M., M.D.*, Assistant Physician to, and Lecturer on Materia Medica at, Guy's Hospital; Licentiate of the Royal College of Physicians; late President of the Westminster Medical Society; Fellow of the Linnean Society of London; Corresponding member of the Philosophical Institute of Basle, of the Philosophical Society of St. Andrews, of the Medical Society of Hamburgh, &c., &c., &c. Philadelphia: Lea & Blanchard. 1845. 8vo. pp. 227.

[CONCLUDED.]

The lungs and kidneys are the great emunctories by which the effete matters of the animal body are eliminated—through the lungs, the matters rich in carbon passing away, in the form of carbonic acid, and by the kidneys, nitrogenised substances, as urea and uric acid, in the human subject. The uric acid of man is replaced in the horse and cow by an acid termed by Liebig the hippuric. Uric acid, according to this eminent chemist, is the first product of the metamorphosed tissues, and becomes urea by a higher oxidation. The acid, having its origin in a deficient supply of oxygen, should be found to exist in excess in diseases which impair the respiratory function, and Liebig states this to be the fact. Some researches, however, in urinary pathology, by Becquerel, render it doubtful. His observations and experiments do not appear to show that there is any fixed relation betwen the urea and uric acid in the urine. In chlorosis, where the blood discs are deficient, and where the urine, in accordance with the theory alluded to, ought to exhibit an excess of uric acid, he found it positively and relatively below the healthy average. In phthisis, again, where we should have looked for the full proportion of urea, and the smallest amount of uric acid, consumption being one of the diseases in which the oxidation of the blood is assumed to be excessive, it appears that instead of being 1 to 31, as in healthy urine, the uric acid was 1 to 7, in one case and 1 to 3⅓ in another.

But we have neither the leisure nor space to pursue these speculative questions, but must refer to the interesting work under review for a satisfactory discussion of them. Excluding all theories regarding the transformation of one excretion into another, we are authorised, in the view of our author, to infer, whenever we find an excess of uric acid or its combinations with bases in the urine, a normal quantity of water being present, (30 to 40 ounces in twenty-four hours,) that one or the other of the following states exists:—

| | |
|---|---|
| A. Waste of tissue more rapid than the supply of nitrogenised nourishment, as in | Fever, acute inflammation, rheumatic inflammation, phthisis. |
| B. Supply of nitrogen in the food greater than is required for the reparation and supply of tissue, as in | Excessive indulgence in animal food, or the quantity of food remaining the same, with too little bodily exercise. |
| C. Supply of nitrogenised food not being in excess, but the digestive function unable to assimilate it: | All the grades of dyspepsia. |
| D. The cutaneous outlet for nitrogenised excreta being obstructed, the kidney is called upon to compensate for this deficient function. | All or most stages of diseases attended with arrest of perspiration. |
| E. Congestions of the kidneys, produced by local causes. | Blows and strains of the loins, diseases of genital apparatus. |

But the fact most interesting to the physiologist and physician, is the comparative insolubility of uric acid. Whatever prevents the oxidation of this acid, and its consequent conversion into urea, must be unfavorable to health, as it gives a tendency to calculous deposits, and otherwise injuriously acts upon the system, as pointed out by Dr. Bird in the following passage:

"Uric acid and urates may occur in great abundance in the urine, so as to become serious sources of irritation, and then especially become primary objects of attention as definite diseases. These bodies may be deposited in an insoluble form in the kidney or bladder, and aggregating, form a mass, on which, by a kind of imperfect crystalization, great portions of the acid or its salts may be deposited, giving rise to the formation of a calculus. Uric acid is of more serious importance than most other elements of calculous formations, not only from its constituting a large proportion of all urinary calculi, but even when they are chiefly composed of other ingredients, the nuclei on which they are deposited are, in the great majority of cases, composed of uric acid. In 374 calculi, contained in the museum of Guy's Hospital the nuclei were in 269 composed of uric acid or urate of ammonia alone."

Uric acid does not always form a deposit when it exists in excess, nor does a deposit necessarily indicate the existence of an abnormal proportion of it. It is not difficult to discriminate between these cases, for, in the words of our author—

"If a deposit of urate of ammonia be present whilst the bulk of the urine in twenty-four hours is not much below the average, it is certain that an excess of uric acid exists. But if the bulk of the urine be much below the natural quantity, a deposit may occur simply from there not being sufficient water to hold it in solution. To determine whether an excess does exist, let all the urine passed in twenty-four hours be collected, well shaken, and a given quantity, as about two ounces, mixed in a conical glass vessel with about half a drachm of hydrochloric acid. In six or eight hours crystals of uric acid will be copiously deposited on the sides of the glass; the urine should be decanted and replaced by cold water. By means of a thin spatula or feather, the acid can be detached and collected at the bottom of the vessel. All the water except the last few drops can be readily poured off without losing the precipitate, which can then be removed into a watch-glass, dried, and weighed. This little operation is so easily performed, that it can scarcely be deemed troublesome; and by a simple multiplication sum, the whole amount of uric acid secreted in twenty-four hours can thus be readily ascertained."

Diet exercises a decided influence on the secretion of this acid, as is happily pointed out by Dr. Bird in the following paragraph:

"The copious deposit of urate of ammonia occurring after eating more freely of animal food than is required for the supply of the wants of the body is a well known phenomenon, and will occur in persons whose digestive organs are in perfect vigor, simply from a greater amount of matter being given them to assimilate than they are adequate to. In like manner, if a person's digestive powers are impaired, either partially or temporarily, as after a debauch, he will be unable to convert into healthy chyle even a small proportion of food, and hence its albuminous elements imperfectly assimilated enter the circulation, to be evolved by the kidneys and perhaps other emunctories. Particular idiosyncrasies with regard to the action of the stomach on certain articles of diet also exist; thus a single cup of coffee or green tea will, in many persons, determine the formation of a deposit in the urine, as if the caffein present in these two beverages had escaped the digestive powers of the stomach, and become converted into urate of ammonia."

The uric acid deposits are recognized by the following characters:

"When heated in the urine, the uric acid deposit does not dissolve; the crystals merely become opaque. It generally becomes more distinct from the solution of urate of ammonia, which is frequently mixed with it, and sometimes completely conceals it from view. Hence the best mode of discovering this deposit, is to warm urine, turbid from excess of urate of ammonia, in a watch-glass; the acid becomes visible in the centre of the glass, as soon as the urate dissolves. Heated with liquor potassæ, the uric acid deposit dissolves, from the formation of an urate of potass of sparing solubility. Hydrochloric and acetic acids are without any action, but the nitric readily dissolves it, and by careful evaporation a residue of a beautiful pink color becoming of a rich purple, on being held over the vapor of ammonia, is left. This colored residue is the murexid of Liebig, the purpurate of ammonia of Dr. Prout. Exposed to heat in a platinum spoon, the uric acid deposits burn, evolving an odour of bitter almonds; and finally leave a small quantity of a white ash, which generally contains phosphate of soda or lime."

The two great points, in the treatment of uric acid gravel, are attention to the function of the skin, and restoration of the tone of the digestive organs. Suppression of the cutaneous excretion is found sensibly to increase the uric acid deposit, and the use of diaphoretics to diminish it. The influence of digestion upon the secretion of the acid has been alluded to, and in correcting the morbid action, the function of the stomach must be kept in view. Diet is the most important measure, and of this, our author says, the patient must use such as he can best digest, "it being of greater importance, in the majority of cases, to regard this, than to choose articles of food according to their chemical composition."

Of remedies which act as solvents of uric acid Dr. Bird makes mention of a number; among the rest, of soda and potassa, and, in the following terms, of phosphate of soda:

"The remarkable solvent action of phosphate of soda on uric acid, to which Liebig has lately directed attention, inspires a hope that its administration might be of use in cases of calculous disease, by impregnating the urine with an active solvent. All that is required to ensure this drug reaching the urine is to administer it in solution sufficiently diluted; ℈j. to ʒss. might be administered in any vehicle, as in broth or gruel, as when diluted, the phosphate tastes like common salt, and few persons object to its flavor. I have administered this drug in two very chronic cases of uric acid gravel, and in one with the effect of rapidly causing a disappearance of the deposit. This occurred in the person of a lady about forty years of age, who had, at my wish, for some weeks used the artificial Vichy water of the German Spa at Brighton without relief. The triple salt, ammonio-phosphate of soda, would perhaps be a more active remedy than the simple phosphate, but its disagreeable flavor constitutes one objection to its employment."

The benzoic acid appears to have some claims upon the practitioner as a solvent of uric acid deposits. We quote our author's remarks concerning it:

"Much attention has been lately drawn to the effects of benzoic acid in preventing the formation of uric acid, by the observations of Mr. Alexander Ure. When this acid or its salts are administered, they are acted upon by the stomach in a very different manner from the other vegetable acids. Instead of becoming oxidized, and being converted into carbonic acid, it combines with those nitrogenised elements which would otherwise have formed urea or uric acid, and is converted into hippuric acid. It has been stated that the quantity of uric acid falls, when the benzoic acid is administered, below the average quantity, or even disappears from the urine. This has been, however, shown by Dr. Garrod, to be an error, and that urea alone disappears. Be this as it may, it is certain that the acid does appropriate to itself some body rich in nitrogen to form hippuric acid; and experience has shown that, in cases where an excess of uric acid is secreted, the administration of this drug appears to limit it to about the normal quantity."

He prescribes it in doses of eight or ten grains in syrup, or dissolved in a weak solution of carbonate or phosphate of soda thrice a day. The following formula he has found of great service in several cases of chronic uric acid gravel:

℞. Sodæ Carbonatis, ʒjss.

Acidi Benzoici, ℈ij.

Sodæ Phosphatis, ʒiij.

Aquæ Ferventis, f℥iv. solve et adde.

Aquæ Cinnamomi, f℥vjiss.

Tincturæ Hyosciami, fʒiv.

Fiat mistura, cujus sumet æger, coch. ij. amp. ter in die.

The next form of urinary deposit mentioned by our author is the *uric oxide*, termed by others *xanthic oxide*, which, however, is so rare a constituent of calculous concretions that we need not stop to speak particularly respecting it. The resemblance between this oxide and the uric acid is so close that much care is required to distinguish between the two deposits; but this is a matter of the less consequence since the indications of cure are the same in each.

*Purpurine* is another pathological product of the kidneys, and is worthy of notice on account of the serious lesions of the liver or spleen, which its presence frequently indicates. It is so soluble in water that it never occurs as a deposit in urine, unless urate of ammonia is present; when this is the case, the ammonical salt assumes a more or less deep carmine tint. To appreciate the beauty of these tints, the deposite must be dried on a filter. Its appearance when so collected, together with its giving up the purpurine to alcohol, will distinguish it from blood, for which it has occasionally been mistaken. The characters of urine containing purpurine are thus given:

"It invariably happens that when an excess of urate of ammonia is present, it, on the urine cooling, falls to the bottom of the vessel, carrying down with it great part of the purpurine. If this excess be not present, the urine simply presents a pink or purple color, and on dissolving white and pure urate of ammonia in it by heat, it is precipitated on cooling deeply colored by the purpurine. If a small portion of purpurine only is present, it is best detected by adding a little hydrochloric acid to some of the urine previously warmed, a color varying from lilac to purple, according to the quantity of coloring matter present, immediately occurs.

"On evaporating urine containing purpurine to the consistence of an extract, and digesting it in alcohol, a fine purple tincture is obtained, the intensity of the tint being rather heightened by acids and diminished by alkalies.

"The specific gravity of this urine is subject to great variation: when the color is as deep as brandy, its density varies from about 1.022 to 1.030. The addition of nitric acid generally produces an immediate muddy deposit of uric acid, which has been more than once mistaken for albumen."

Another substance, now and then found in urinary sediments, and which enters into the composition of a rare species of calculus, is *cystine*, the diagnosis of which is fully pointed out in the volume of Dr. Bird. The urine containing it is very turbid, and soon deposits a copious sediment, not affected by heat, and only slowly dissolved in nitric or muriatic acid; insoluble in the vegetable acids, but soluble in the fixed alkalis and their carbonates, and burning in a platina spoon with a peculiar and disagreeable odor. From urate of ammonia it is distinguished by not disappearing on warming the urine, and from the earthy phosphates by being insoluble in dilute muriatic or strong acetic acid.

The treatment in cases of this morbid secretion is not well understood. Most is to be done by attention to the health of the assimilative functions.

Dr. Bird has heen led to question the accuracy of the opinion, according to which *oxalate of lime* is a very rare urinary deposit. He has met with it frequently, and holds it to be connected with certain nervous maladies which abound in populous cities. The digestive organs are deranged in those individuals who afford this deposit, and it is strikingly increased or diminished by particular kinds of food. It is, therefore, especially amenable to dietetic regulations. The symptoms attending it are described in the following words:

"Persons affected with the disease under consideration are generally remarkably depressed in spirits, and their melancholy aspect has often enabled me to suspect the presence of oxalic acid in the urine. I have seldom witnessed the lurid greenish hue of the surface to which Dr. Prout has referred. They are generally much emaciated, excepting in slight cases, extremely nervous, and painfully susceptible to external impressions, often hypochondriacal to an extreme degree, and in the majority of cases labor under the impression that they are about to fall victims to consumption. They complain bitterly of incapability of exerting themselves, the slightest exertion bringing on fatigue. In temper they are irritable and excitable; and in men the sexual power is generally deficient, and often absent. A severe and constant pain, or sense of weight, across the loins, is generally a prominent symptom. The mental faculties are generally but slightly affected, loss of memory being sometimes more or less present. Well-marked dyspeptic feelings are always complained of. Indeed, in most of the cases in which I have been consulted, I have been generally told that the patient was ailing, losing flesh, health, and spirits, daily; or remaining persistently ill and weak, without any definite or demonstrable cause."

We come next to the consideration of the *earthy salts* of urine—phosphate of lime, ammonio-phosphate of magnesia, and carbonate of lime, the account of which in this volume is full and instructive. They occur in consequence of a deficiency of acid in the urine, as carbonate of lime is deposited on the escape of carbonic acid halding it in solution, and, indeed, it seems that "where phosphate of lime forms the great bulk of a deposit, a certain portion of carbonate is generally present."

The details on this subject are too numerous to be embodied in the limits within which we must confine ourselves, and for all that relates to the diagnosis and treatment we must therefore refer our readers to the work itself.

Until lately, *carbonate* of lime, although of constant occurrence in the urine of herbivorous animals, was not believed to enter into the composition of any urinary calculi in the human subject; but it turns out, that this substance often occurs in small quantities mingled with the earthy phosphates, and is more apt to be present when the urine is decidedly alkaline. Its origin, according to Dr. Bird, "may then be explained by a decomposition of phosphate of lime by the carbonate of ammonia which replaces the urea." It then appears simply as an amorphous powder, and may easily be recognized by its effervescence with any dilute acid, the carbonate of ammonia having been carefully washed out by warm water.

The urine is subject to discoloration, not only by blood and bile, but by various products of diseased action, among which are enumerated, cyanourine, indigo, per-cyanide of iron, melanourine, and melanic acid, from which it receives blue or black tints more or less intense. But as the presence of such matters is not frequent their diagnosis and pathology need not detain us.

The following chapter is one of the most interesting in the volume, embracing *non-crystalline organic deposits*, such as albumen, hæmatosine, pus, mucus, milk, fatty matters, &c. These substances are to be detected by the microscope, other tests being comparatively unavailing. This remark, however, is not applicable to *albumen*, which gives an opacity to urine on the application of heat. If it be abundant, the urine will become quite solid when so treated, and the opalescence will vary according to the quantity present in the urine. Nitric acid also coagulates albumen, and if these tests concur, its presence is no longer doubtful. The two ought always to be applied in conjunction.

When blood is present in urine, that fluid is more or less discolored, according to the quantity, and as albumen is one of the constituents of the blood, it will be found that the urine under such circumstances is coagulable by heat and nitric acid; but in order to render the diagnosis clear, in doubtful cases, the microscope must be applied. In reference to the treatment of hæmaturia Dr. Bird says—

"No remedy has, however, appeared to me to be of such extraordinary value in the treatment of hæmaturia as gallic acid. I have seen this drug arrest for many weeks bleeding from an enlarged (and fungoid?) kidney, after all other remedies had failed. It should be given in doses of five grains in a draught, with mucilage, and a little tinct. hyoscyami, and repeated at short intervals. This drug really acts as a direct astringent, reaching the capillaries of the kidney, and finding its way into the urine, which soon becomes so impregnated with it, as to be changed into ink on the addition of a few drops of tinctura ferri sesqui-chloridum."

Milk, it appears, has never been detected in the urine as a morbid element, but is sometimes present as a fraudulent admixture. The milk-like urine often seen, derives its appearance from phosphatic, purulent, or fatty secretions mingled with it. Still, Dr. Bird concedes that some of the ingredients of milk may be contained in the urine of pregnant women, having been taken into the circulation from the mammary glands. *Kiestein* is the name proposed for the matter detected in such cases; and *gravidine* has also been suggested, but Dr. Bird is not satisfied of its being a distinct organic principle.

*Spermatozoa* are by no means very unfrequent in urinary deposits, in some cases imparting to the urine their characteristic odor.

"Should a larger quantity of the secretion be present, it subsides to the bottom of the vessel, and may be recognized by its physical character. If mere traces of spermatic liquor only are mixed with urine, they may easily be detected by violently agitating, and allowing it to repose in a conical glass vessel for a few hours. On carefully decanting all the urine except the last few drops, the spermatozoa may be detected in the latter by the microscope."

From a series of observations recently made, there is strong reason to believe that fermentation is attended, if not produced, by the appearance of certain confervoid or fungoid vegetations, which pass through regular stages of development. When the urine contains sugar these vegetations make their appearance. The account given by our author of these appearances is highly curious. He states that—

"When saccharine urine is left in a warm place, a scum soon forms on its surface, as if a little flour had been dusted upon it. This consists of minute oval bodies which soon enlarge from the development of minute granules visible in their interior. These continue expanding, and dilate the oval vesicle containing them into a tubular form; soon afterwards the internal granules become larger and transparent, and project from the exterior of the parent vesicle like buds. The whole then resembles a jointed confervoid growth, which ultimately breaks up; and a copious deposit, of oval vesicles or spores, falls to the bottom. All these stages of development require but a few hours for their completion. If the deposited spores be placed in weak syrup they rapidly germinate, and exciting fermentation, produce a new crop of torulæ. During the growth of the torula, bubbles of carbonic acid gas are evolved, and the urine at length acquires a vinous odor, sometimes accompanied by an odor of butyric acid."

And, "still they come;" not only *torulæ*, these little mushroom plants, but *vibriones*, minute animalcules, "are occasionally developed in the urine, so soon after passing as to lead to the idea that their germs must have existed in the urine whilst in the bladder."

In the concluding chapter, at which we have arrived in our cursory notice, Dr. Bird makes some judicious practical remarks on the therapeutical employment of remedies influencing the functions of the kidneys. These remedies have been often complained of by writers as capricious and uncertain in their action, and the complaint is just, as practitioners too well know. But there is a cause for this variableness in the action of diuretics, and without stopping to inquire into the nature of it, we quote from our author certain laws deduced from the recorded experience of the medical profession, the observance of which will enable us to predict with a great degree of certainty what will be the effect of these therapeutic agents:

"*Law 1st.* All therapeutical agents intended to reach the kidneys must either be in solution when administered, or capable of being dissolved in the fluids contained in the stomach or small intestines, after being swallowed."

"*Law 2d.* Bodies intended to reach the kidneys must, to ensure their absorption, have their solutions so diluted as to be of considerably lower density than either the liquor sanguinis, or serum of blood (i. e. below 1.028)."

"*Law 3d.* 'If a sufficent quantity of water cannot be received into the small intestines, or the circuit through the portal system in the vena-cava ascendens, or thence through the lungs and heart into the systemic circulation, be obstructed, or if there be extensive disorganisation of the kidneys, the due secretion of urine cannot be effected.' "

The following practical conclusions should be appended to the foregoing laws:

"1. Whenever it is desirable to impregnate the urine with a salt, or to excite diuresis by a saline combination, it must be exhibited in solution, so diluted as to contain less than 5 per cent. of the remedy, or not more than about twenty-five grains in an ordinary draught. The absorption of the drug into the capillaries will be ensured by a copious draught of water, or any diluent, immediately after each dose.

"2. When the urine contains purpurine, or other evidence of portal obstruction exist, the diuretics or other remedies employed should be preceded or accompanied by the administration of mild mercurials,—taraxacum, hydrochlorate of ammonia, or other cholitic remedies. By these means, or by local depletion, the portal vessels will be unloaded, and a free passage obtained to the general circulation.

"3. In cases of valvular or other obstructions existing in the heart and large vessels, it is next to useless to endeavor to excite diuretic action, or appeal to the kidneys by remedies intended to be excreted by them. The best diuretics here will be found in whatever tends to diminish the congested state of the vascular system, and to moderate the action of the heart; as digitalis, colchicum, and other sedatives, with mild mercurials."

The reader who wishes to study the subject of urinary deposits by the best light of modern science, will avail himself of the aid offered in the pages of this work. It must take precedence of all the treatises on that subject.

